# The FRK1 mitogen-activated protein kinase kinase kinase (MAPKKK) from *Solanum chacoense* is involved in embryo sac and pollen development

**DOI:** 10.1093/jxb/eru524

**Published:** 2015-01-08

**Authors:** Edith Lafleur, Christelle Kapfer, Valentin Joly, Yang Liu, Faiza Tebbji, Caroline Daigle, Madoka Gray-Mitsumune, Mario Cappadocia, André Nantel, Daniel P. Matton

**Affiliations:** ^1^Institut de recherche en biologie végétale, Département de sciences biologiques, Université de Montréal, 4101 rue Sherbrooke est, Montréal, QC H1X 2B2, Canada; ^2^Institut de recherche en biotechnologie, Conseil national de recherches du Canada, 6100 Avenue Royalmount, Montréal, QC H4P 2R2, Canada

**Keywords:** Embryo sac development, gametophyte to sporophyte communication, MAPKKK, megagametogenesis, microgametogenesis, pollen tube guidance, seed and fruit development, *Solanaceae*.

## Abstract

The fertilization-related kinase 1 (ScFRK1), a nuclear-localized mitogen-activated protein kinase kinase kinase (MAPKKK) from the wild potato species *Solanum chacoense*, belongs to a small group of pMEKKs that do not possess an extended N- or C-terminal regulatory domain. Initially selected based on its highly specific expression profile following fertilization, *in situ* expression analyses revealed that the *ScFRK1* gene is also expressed early on during female gametophyte development in the integument and megaspore mother cell and, later, in the synergid and egg cells of the embryo sac. *ScFRK1* mRNAs are also detected in pollen mother cells. Transgenic plants with lower or barely detectable levels of *ScFRK1* mRNAs lead to the production of small fruits with severely reduced seed set, resulting from a concomitant decline in the number of normal embryo sacs produced. Megagametogenesis and microgametogenesis were affected, as megaspores did not progress beyond the functional megaspore (FG1) stage and the microspore collapsed around the first pollen mitosis. As for other mutants that affect embryo sac development, pollen tube guidance was severely affected in the *ScFRK1* transgenic lines. Gametophyte to sporophyte communication was also affected, as observed from a marked change in the transcriptomic profiles of the sporophytic tissues of the ovule. The ScFRK1 MAPKKK is thus involved in a signalling cascade that regulates both male and female gamete development.

## Highlight

This study reveals the ScFRK1 MAP kinase kinase kinase as a novel player in male and female gametophyte development, ultimately affecting pollen tube guidance and gametophyte to sporophyte communication.

## Introduction

Flowering plants or angiosperms exhibit a two-staged life cycle, alternating between a short-lived haploid gametophyte generation composed of only a few cells, and a temporally predominant diploid sporophytic generation. The haploid generation begins with specialized diploid cells (mother cells) of the sporophyte that undergo meiosis to give rise to haploid spores. These spores undergo cell proliferation and differentiation to produce multicellular haploid gametophytes. The male gametophyte (pollen grain) develops within the stamen of the anther and consists of two sperm cells encased within a vegetative cell. The female gametophyte (embryo sac or megagametophyte) develops within the carpel of the ovary and, in most cases, leads to the formation of an eight-nucleate, seven-celled *Polygonum*-type embryo sac harbouring one egg cell, two synergid cells, three antipodal cells, and one central cell. The major function of gametophyte generation is thus to produce haploid gametes, the egg and sperm cells, which, upon fusion (one sperm cell fusing with the egg cell and the other with the central cell, giving rise to the embryo and endosperm, respectively), will lead to a new sporophytic generation.

Numerous genetic screens have been carried out to identify genes affecting gametophyte development (Feldmann *et al.*, 1997; Bonhomme *et al.*, 1998; Christensen *et al.*, 1998; Pagnussat *et al.*, 2005; Muralla *et al.*, 2011). One would expect that, considering the developmental complexity involved in gamete production, genes involved in intracellular signalling and cell–cell communication would be readily found among gametophyte essential genes. From the curated data set of *Arabidopsis* genes required for gametophyte function listed in Muralla *et al.* (2011), of the 173 genes that displayed a gametophyte defective phenotype, only 9 (0.05%) are classified as being involved in signalling pathways, of which three are protein kinases. These include the two-component histidine kinase CKI1 (At2g47430) involved in cytokinin perception that severely affects the female gametophyte but only weakly affects the male gametophyte (Pischke *et al.*, 2002; Hejatko *et al.*, 2003); the FUSED (FU) Ser/Thr kinase, involved in cytokinesis, that severely affects both male and female gametophytes (Oh *et al.*, 2005); and the calmodulin-binding receptor-like cytoplasmic kinase 2 (CRCK2) that severely affects male gametophyte development but only weakly affects the female gametophyte (Boavida *et al.*, 2009).

With >1000 and 1400 members, respectively, the kinase superfamily from *Arabidopsis* and rice represents a very large fraction of the proteome in comparison with other eukaryotes. For example, in *Arabidopsis*, kinases represent 4% of the proteome, compared with ~2% in human, *Caenorhabditis elegans*, *Drosophila melanogaster*, and yeast (Shiu and Bleecker, 2003; Shiu *et al.*, 2004; Champion *et al.*, 2004; Dardick *et al.*, 2007). Nonetheless, only a few kinases have been found in gametophyte defective mutant screens, most probably due to the high level of functional redundancy found in major kinase groups, mainly the receptor kinase family (>600 in *Arabidopsis* and 1200 in rice) and the mitogen-activated protein kinase (MAPK) superfamily (MAPK, MAPKK, MAPKKK, and MAPKKKK) with >100 members in both *Arabidopsis* and rice (Hamel *et al.*, 2006; Rao *et al.*, 2010). An example of such redundancy in the MAPKKK family is observed with the *Arabidopsis* ANP1/2/3 kinases that regulate cell division where the triple mutant is not transmitted through the male and female gametes, although other phenotypes, such as reduction of plant size (*anp2*/*anp3* double mutants), were also observed (Krysan *et al.*, 2002). The ANP1/2/3 kinases are related to the tobacco NPK1 MAPKKK that is part of a cascade, the NACK–PQR pathway, possibly involved in cellularization/differentiation which occurs during stage FG5 (Nishihama *et al.*, 2002; Soyano *et al.*, 2003; Chevalier *et al.*, 2011).

In this study, the isolation and functional characterization of a new MAPKKK from the pMEKK subfamily in *Solanum chacoense* is described. Down-regulation of this single MAPKKK named ScFRK1 (fertilization-related kinase) severely affects both embryo sac and pollen development and leads to partial parthenocarpic fruit production upon pollination.

## Materials and methods

### Plant material and plant transformation

All plant material and growth conditions are as described in Gray-Mitsumune *et al.* (2006). For sense and antisense constructs, the *ScFRK1* cDNA was inserted in a modified pBin19 transformation vector with a *Cauliflower mosaic virus* (CaMV) 35S double enhancer promoter (Bussière *et al.*, 2003). Sense and antisense constructs were individually transformed in *Agrobacterium tumefaciens* LBA4404 by electroporation. *Solanum chacoense* plants were transformed by the leaf disc method as previously described (Matton *et al.*, 1997).

### DNA and RNA analyses

Nucleic acid isolation, blotting, and hybridization are as described in O’Brien *et al.* (2007). Sequence analysis and phylogeny are as described previously (Gray-Mitsumune *et al.*, 2006). Accession numbers are AY427828, KC768863 (*ScFRK1*), AY427829 (*ScFRK2*), and KC768864 (*ScFRK3*).

### Protein localization through transient expression

The *ScFRK1* coding region was fused in-frame to the N-terminus of green fluorescent protein (GFP) in the 35S-driven Gateway vector pMDC83 (Curtis and Grossniklaus, 2003). A 35S::GFP construct was used as a control. Microparticle bombardment was performed as described previously (Germain *et al.*, 2008).

### Tissue fixation and electron microscopy observations

For pollen viability estimation through outer structure analysis, fresh pollen was observed with a Hitachi S-3000N variable pressure scanning electron microscope at 30 Pa and 15kV. For transmission electron microscopy (TEM), samples were fixed in 2.5% glutaraldehyde in 0.1M sodium cacodylate buffer pH 7.4, post-fixed in 2% osmium tetroxide (OsO_4_) in the same buffer, dehydrated in ethanol, and embedded in Spurr’s resin. Observations were made on a Hitachi H-7500 microscope. Statistics for pollen defects observed by scanning electron microscopy (SEM) were scored from ≥100 pollen grains per wild-type (WT) or transgenic line.

### Cytological analysis of microsporogenesis

Meiosis and microspore development were studied by squashing anthers in lacto-acetic orcein (1% orcein), according to Dyer’s method (Dyer, 1963), modified by substituting propionic acid with acetic acid. To monitor starch accumulation inside developing pollen, anthers were also squashed in iodine (I_2_–KI), according to Eriksson’s method (Eriksson, 1962). Pollen fertility was estimated after staining freshly collected mature pollen with acetocarmine (1%) or iodine. All observations were made with a Zeiss AxioImager M1 microscope equipped with an AxioCam HRc camera.

### Tissue fixation and optical microscopy observations

Pistils were fixed in FAA for 24h at 4 °C (50% ethanol, 1.35% formaldehyde, and 5% glacial acetic acid). Samples were then dehydrated in increasing series of *tert*-butyl alcohol (from 70% to pure *tert*-butyl alcohol). Pistils were infiltrated with Paraplast Plus paraffin at 60 °C. Thin sections (10 μm) were prepared from embedded samples and tissue sections were stained in 0.5% Astra Blue and 1% safranine after paraffin removal. Alternatively, thin sections (10 μm) were prepared from embedded samples and tissue sections were stained in 0.05% Toluidine Blue. *In situ* hybridizations were performed as described previously (O’Brien *et al.*, 2005). For differential interference contrast (DIC) microscopy observations, floral buds were dissected and ovaries were fixed in FAA solution overnight (50% ethanol, 0.5% acetic acid, and 1% formaldehyde). Clearing of ovules was performed with an increasing ratio of ethanol–methylsalicylate solutions (0:100, 75:25, 50:50, 25:75, 100:0) for 1h each and left overnight in 100% methylsalicylate. After dissection from the placenta, ovules were observed with a Zeiss AxioImager M1 microscope equipped with an AxioCam HRc camera.

### cDNA microarrays analysis

DNA microarrays comprised 7741 expressed sequence tags (ESTs) corresponding to 6374 unigenes derived from fertilized ovary cDNA libraries covering embryo development from zygote to late torpedo stages (Germain *et al.*, 2005). Experimental conditions were as described previously (Gray-Mitsumune *et al.*, 2006; Tebbji *et al.*, 2010).

## Results

### Sequence analysis and cellular localization of the ScFRK1 kinase

Using a subtraction selection screen targeting only genes weakly expressed during fertilization and early embryogenesis, five members from the MAPKKK family were isolated in *S. chacoense*, a self-incompatible wild potato species (Germain *et al.*, 2005). Three of these, named *ScFRK1*– *ScFRK3*, were phylogenetically classified in the pMEKK subfamily of the MAPKKKs (Gray-Mitsumune *et al.*, 2006), although they differed significantly from the majority of the pMEKKs due to their small size, consisting of practically only a kinase domain with little N- or C-terminal putative regulatory domains. In *Arabidopsis thaliana*, 21 MAPKKKs are classified as pMEKKs (mean size ~675 amino acids), with five of those (AtMAPKKK17–AtMAPKKK21) <400 amino acids in length. Three of these (AtMAPKKK19–AtMAPKKK21) are closely related to the ScFRK1–ScFRK3 family in *S. chacoense*, although ScFRK3 is closer to the MAPKKK19–MAPKKK21 group (Supplementary Fig. S1A available at *JXB* online). Amino acid sequence identity within this group ranges from 31% to 75% (46–85% similarity) (Supplementary Fig. S1B). Functional analysis of this family has only been reported for the ScFRK2 kinase, which has been shown to be involved in ovule and pollen development (Gray-Mitsumune *et al.*, 2006; O’Brien *et al.*, 2007), and AtMAPKKK20, involved in the osmotic stress response (Kim *et al.*, 2012). Here the functional analysis of the ScFRK1 kinase is reported.

**Fig. 1.**
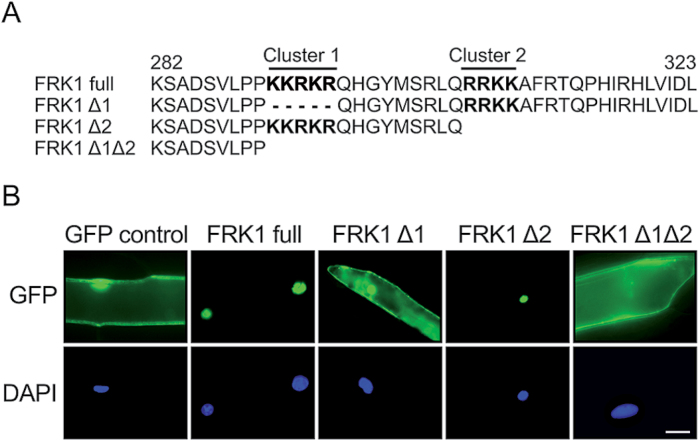
Characterization of the ScFRK1 bipartite nuclear localization signal. (A) Details of the wild type and modified C-terminal region of ScFRK1 constructs used for protein localization studies. The *ScFRK1* coding region was fused in-frame to the N-terminus of GFP. The two short basic amino acid sequences predicted to form the NLS are shown in bold. (B) Visualization of GFP expression (top) and DAPI (nucleus) localization (bottom) in bombarded onion cells expressing the fusion constructs. Scale bar=25 µm.

The *ScFRK1* clone codes for an open reading frame of 323 amino acids with an estimated mol. wt of 37kDa. A short C-terminal region of 42 amino acids (position 282–323, Fig. 1A) follows kinase subdomain XI. Analysis of the sequence revealed the presence of a cluster of two short basic amino acid sequences predicted to form a bipartite nucleoplasmin-type nuclear localization sequence (NLS) (Brameier *et al.*, 2007) (Fig. 1A). The algorithm also predicted a higher NLS potential for the first one (NLS1). To verify this, the ScFRK1 coding region was fused in-frame to the N-terminus of GFP. A 35S::GFP construct was used as a control. As expected for the GFP alone, expression was detected in both the cytoplasm and the nucleus (Fig. 1B; GFP control). In contrast, fluorescence of the ScFRK1–GFP fusion protein was restricted to the nuclei and co-localized with the 4′,6-diamidino-2-phenylindole (DAPI) signal (Fig. 1B; ScFRK1 full). To determine if the two basic sequences were used as a bipartite NLS or if they acted redundantly, the individual role of each predicted NLS was analysed. As shown in Fig. 1B, a sharp cytoplasmic fluorescence was observed in most of the onion cells bombarded with the ScFRK1Δ1 or ScFRK1Δ1Δ2 constructs. However, ambiguous nucleocytoplasmic localization was obtained in ~45% of bombarded cells with the ScFRK1Δ1 construct. Consistent with the above-mentioned prediction, the deletion of NLS2 did not disrupt the nuclear localization of ScFRK1 to the same extent. Only 25% of the cells bombarded with the Δ2 construct showed cytoplasmic or nucleocytoplasmic fluorescence (data not shown). This suggests that although the two C-terminal NLS in ScFRK1 form a bipartite NLS, NLS1 predominates, consistent with the NLS strength prediction.

### Pollination and fertilization trigger a stepwise decrease of *ScFRK1* mRNA abundance in ovaries

**Fig. 2.**
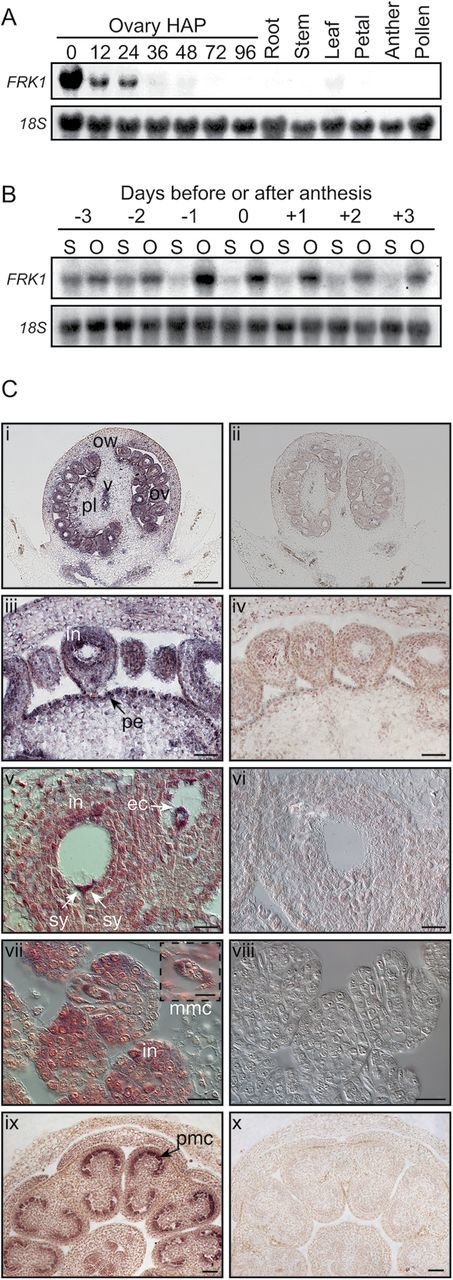
*ScFRK1* expression analysis. (A) RNA gel blot analysis of the *ScFRK1* gene. All tissues were collected from greenhouse-grown plants. Fertilized ovaries were dissected from pistils 0–96 hours after pollination (HAP). A 10 μg aliquot of total RNA isolated from *S. chacoense* tissues was blotted and probed using the full-length *ScFRK1* cDNA (upper panel). Membranes were stripped and re-probed using a partial 18S rRNA to ensure equal loading of each RNA sample (lower panel). (B) RNA gel blot analysis of the *ScFRK1* gene before and after anthesis in unpollinated pistil tissues. S, style; O, ovary. (C) *In situ* localization of *ScFRK1* transcripts in ovules and anthers. (i and ii) Unfertilized mature ovary sections. (iii and iv) Magnification of unfertilized mature ovary sections. (v and vi) *ScFRK1* expression in the ovule integument and embryo sac of mature ovules at anthesis. (vii and viii) *ScFRK1* expression in young ovules at the megaspore mother cell stage isolated from ~2mm flower buds. (ix and x) Cross-sections of young anthers isolated from ~2mm flower buds. i, iii, v, vii, and ix, antisense probe. ii, iv, vi, viii and x, control sense probe. Digoxigenin labelling is visible as red to purple staining. All hybridizations used 10 μm thick sections and an equal amount of either *ScFRK1* sense or antisense probe. ec, egg cell; in, integument; mmc, megaspore mother cell; ov, ovule; ow, ovary wall (pericarp); pe, placenta epidermis; pl, placenta; sy, synergid, zy, zygote. Images v–viii were taken under DIC optics in order to better show the cellular structures. Scale bars: 200 μm (i, ii); 50 μm (iii, iv, ix, x); and 20 μm (v–viii).

The *ScFRK1* expression pattern was determined by RNA gel blot analysis with various vegetative (roots, stems, and leaves), generative (petals), and reproductive tissues (stamens, pollen, styles, and ovaries). At anthesis, strong *ScFRK1* mRNA accumulation was only observed in the ovary (Fig. 2A) and, to a lesser extent, in the style (Fig. 2B). Faint expression could also be detected in the leaf (Fig. 2A). Pollination and fertilization had dramatic effects on *ScFRK1* accumulation in ovaries. Although pollen tubes only reach the ovules ~36h after landing on the stigma, *ScFRK1* steady-state mRNA levels had already significantly declined 12h after pollination and were barely detectable after fertilization (Fig. 2A). To determine if this stepwise down-regulation of *ScFRK1* steady-state mRNA levels was caused by pollination and fertilization, and was not developmentally regulated, non-pollinated pistils were collected from 3 d before anthesis to 3 d after anthesis. As shown in Fig. 2B, peak accumulation of *ScFRK1* mRNAs is observed 1 d prior to anthesis and, without pollination, only slightly declines in ovary thereafter. Even 3 d after anthesis, strong *ScFRK1* mRNA accumulation is still observed, confirming the stepwise roles of pollination and fertilization in *ScFRK1* mRNA accumulation in ovaries.

### *ScFRK1* is expressed in both the sporophyte and the gametophyte

In order to determine the spatial expression pattern of the *ScFRK1* gene, *in situ* RNA hybridizations were performed using gynoecia from various developmental stages (Fig. 2C). On the day of anthesis, *ScFRK1* mRNA signal was strongly detected in ovules and in the vascular tissue and, to a lesser extent, in the ovary wall (Fig. 2C, i). At medium magnification, the strongest accumulation is detected in the integument of the ovule as well as in the epidermis of the placenta that is in direct continuity with the ovule integument (Fig. 2C, iii). Closer examination of the *ScFRK1* expression pattern revealed that the gene is expressed in the synergids and the egg cell of the embryo sac (Fig. 2C, v). The asymmetric staining pattern observed is typical for these cells since the two synergids have their large vacuole located towards the chalazal pole, while the vacuole of the egg cell has the reverse orientation. This concentrates the mRNA signal at the micropylar pole for the synergids and towards the chalazal pole for the egg cell. In young flower buds bearing ovules at the megaspore mother cell (MMC) stage, *ScFRK1* mRNA signal was already observed in the single ovule integument (Fig. 2C, vii). *Solanum chacoense* produces unitegmic-tenuinucellate ovules, a trait that occurs almost universally in the asterid clade (Albach *et al.*, 2001). At this stage, denser staining was consistently observed at the tip of the growing integument as well as in the MMC (Fig. 2C, vii). At the same stage, *ScFRK1* mRNA signal was also detected in developing anthers, more prominently observable in pollen mother cells (PMCs; Fig. 2C, ix).

### *ScFRK1* knock-down transgenic lines show reproductive defects

**Fig. 3.**
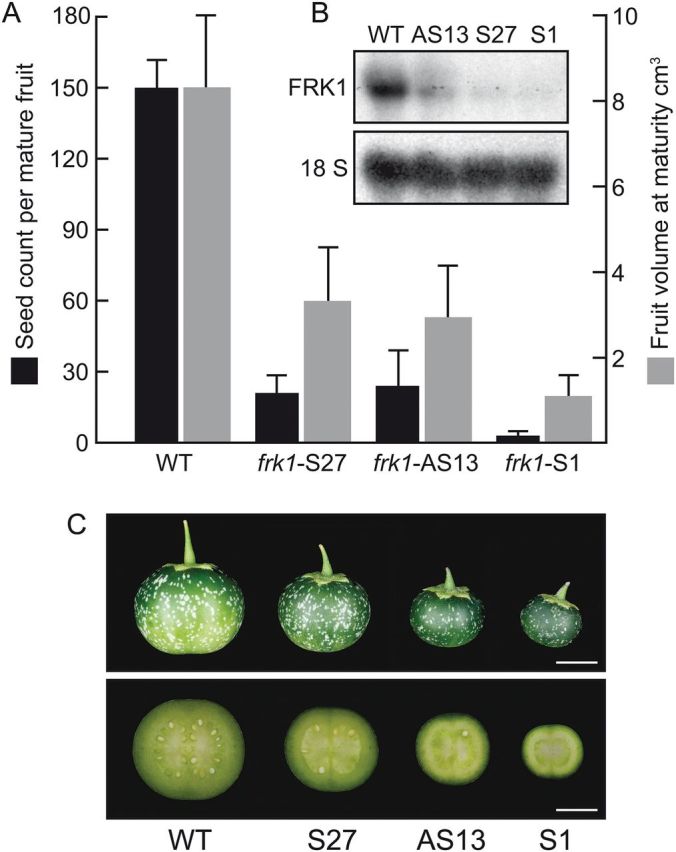
Analyses of *ScFRK1* transgenic plants. (A) Seed count and fruit volume measurements from WT plants and transgenic lines S1, S27, and AS13. (B) RNA gel blot analyses of *ScFRK1* mRNA (upper panel) and 18S rRNA (lower panel) of ovary tissues derived from WT plants and transgenic lines S1, S27, and AS13. (C) Comparison of fruits and fruit slices from WT plants and transgenic lines S1, S27, and AS13. Scale bar=1cm. (This figure is available in colour at *JXB* online.)

In order to assign a function to the *ScFRK1* gene, transgenic plants carrying an *ScFRK1* sense or antisense construct were generated. The *ScFRK1* cDNA was placed downstream of a double enhancer CaMV35S promoter in a modified pBin19 vector in a sense or antisense orientation (Bussière *et al.*, 2003). Kanamycin-resistant plants were grown to maturity in the greenhouse and cross-pollinated to determine if any abnormal phenotype linked to sexual reproduction, based on the *ScFRK1* expression profile, could be observed. Numerous plants showed a marked reduction in fruit size, irrespective of the transgenic population from which they were isolated (sense or antisense lines). The *ScFRK1* expression level was monitored in transgenic lines showing a decrease in fruit size by RNA gel blot analyses of ovaries collected on the day of anthesis. All the affected lines showed a reduced accumulation of *ScFRK1* mRNAs. Three lines expressing variable levels of *ScFRK1*, down to almost undetectable levels, were chosen for further analyses (Fig. 3). Lines S27 and S1 were co-suppressed lines retrieved from the *ScFRK1* sense overexpression population, while AS13 came from the antisense expressed population. Overall plant growth and vegetative development appeared unaffected in all *ScFRK1* transgenic lines. However, the *ScFRK1* down-regulated lines exhibited severe defects in seed and fruit development. Fruit volume ranged from 13% (S1) to 35–40% (AS13 and S27) when compared with the WT (Fig. 3A, C) or transgenic plants unaffected in *ScFRK1* expression (data not shown). Seed production was also strongly affected, with S1 producing only 2% of the normal seed content of an *S. chacoense* fruit, while AS13 and S1 produced only 15% of the WT seed count (Fig. 3A). The reduced seed set could thus explain the small fruit size observed.

### Down-regulation of *ScFRK1* affects embryo sac development

**Fig. 4.**
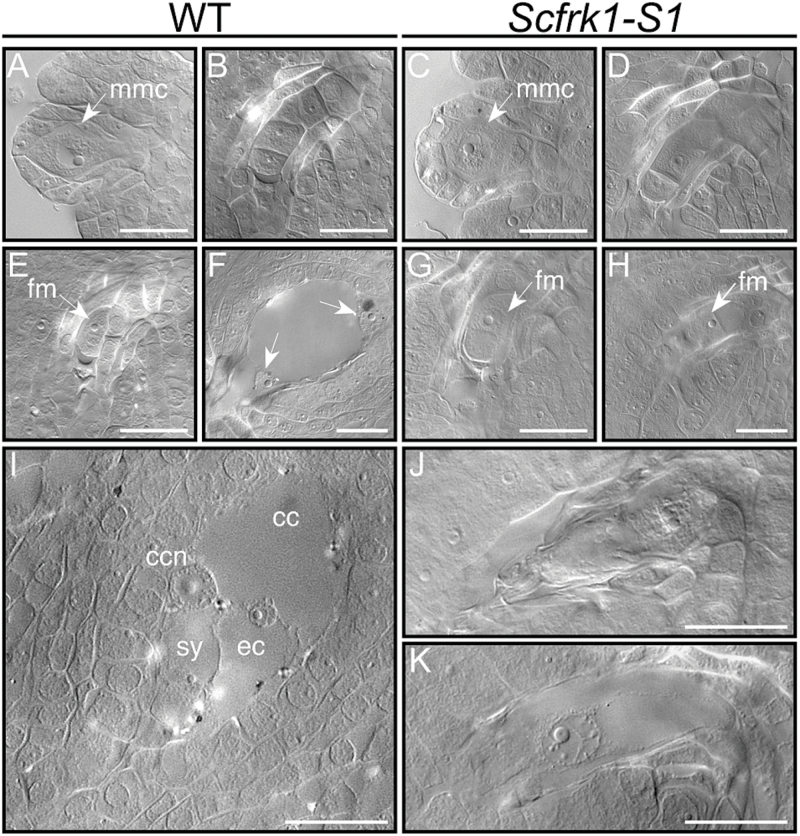
Megasporogenesis and megagametogenesis in WT *S. chacoense* and in the *Scfrk1*-S1 transgenic plant line. Cleared ovules were observed by DIC microscopy. Stages determined following Table 2. (A, C) Megaspore mother cell stage. (B, D) Tetrad stage. (E, G) Functional megaspore stage (FG1). (F, H) Binucleated megagametophyte stage. In F, the arrows point to the two nuclei in the late two-nucleate stage (FG3). In H, embryo sac development is halted at the FG1 stage in the *Scfrk1*-S1 transgenic line. (I, J, K) Mature embryo sac stage at anthesis (FG8). mmc, megaspore mother cell; fm, functional megaspore; cc, central cell; ccn, central cell nucleus; sy, synergid cell; ec, egg cell. Scale bar=20 μm.

Since *ScFRK1* is expressed before and after anthesis and with an expression level influenced by both pollination and fertilization, the reduced seed set observed could result from either aberrant ovule development or post-fertilization seed abortion. To assess this, cleared ovules from all lines were observed prior to pollination. As shown in Table 1, decreasing levels of *ScFRK1* mRNA led to a concomitant decrease in the number of normal embryo sacs observed. In the most strongly affected transgenic line, *Scfrk1*-S1, this led to an almost complete absence of normal embyro sacs, explaining the strongly reduced seed set observed. In order to determine when the defect appeared during female gametophyte development, ovules from different developmental stages were observed in flowers buds of the *Scfrk1*-S1 line, since almost all of its ovules were affected at anthesis. Table 2 shows the correspondence between flower bud length and developmental stages of the *S. chacoense* female gametophyte. Observation of cleared ovules revealed that megasporogenesis was unaffected. Ovules from the MMC to the functional megaspore stage could be routinely observed in both WT plants (Fig. 4A, B, E) and the *Scfrk1*-S1 line (Fig. 4C, D, G). Thus, meiosis of the MMC ultimately producing the functional megaspore appeared normal in the *ScFRK1* transgenic line. Afterwards, no cell divisions could be observed in the *Scfrk1*-S1 line (Fig. 4H that would correspond to the dyad stage in the WT shown in Fig. 4F, and later at anthesis in Fig. 4J or K). In the WT plants, the surviving megaspore underwent three successive mitotic divisions to produce an eight-nucleate megagametophyte. In WT ovules at anthesis, the three antipodals have already degenerated and only the central cell with its fused polar nuclei, the synergids, and the egg cell are visible (Fig. 4I). In the transgenic lines, affected embryo sacs showed either a clear lack of organization with a shrunken and filled embryo sac (Fig. 4J) or retained a single cell (Fig. 4K). Thus, down-regulation of the *ScFRK1* gene affects megagametogenesis as the functional megaspore never progresses beyond the FG1 stage.

**Table 1.** Percentage of normal and abnormal embryo sacs as observed in cleared ovules by DIC microscopy CC, central cel; EC, egg cell; ES, embryo sac; SY, synergid. Fifty ovules were observed for each plant line in two consecutive generations.

Plant line	Normal ES	Modified ES	ES absent
WT	96	4	0
*frk1*-AS13	44	30	26
*frk1*-S27	32	22	46
*frk1*-S1	0	6	94
	ES with 1 EC, 2 SY, and 1CC	ES with fewer cells	No ES observed

**Table 2.** Correspondence between flower buds length and developmental stages of *S. chacoense* female gametophyte

Flower bud length	Female gametophyte development stage
1.0–1.5 mm	Ovule primordia
1.5–2.5 mm	Megaspore mother cell
2.5–3.0 mm	Dyad
3.0–4.0 mm	Tetrad and functional megaspore
4.0–5.0 mm	Uninucleated and binucleated embryo sac
5.0–6.0 mm	Tetranucleated and octanucleated embryo sac
Open flower–anthesis	Mature embryo sac four nuclei (antipodals have degenerated)

Abbreviations: FRK, fertilization-related kinase; MAPK, mitogen-activated protein kinase; MAPKK, mitogen-activated protein kinase kinase; MAPKKK, mitogen-activated protein kinase kinase kinase; WT, wild type.

### Pollen development is also affected in *ScFRK1* transgenic lines

**Fig. 5.**
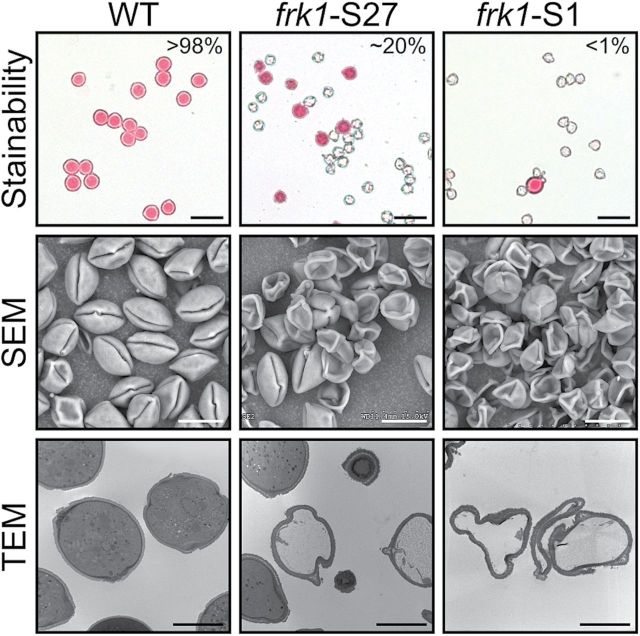
Pollen viability estimation in WT and two *ScFRK1* transgenic plant lines. Pollen viability and phenotype were estimated through viable stain analysis and electron microscopy observations. Upper panels: pollen was stained with 1% acetocarmine. Viable pollen grains are lightly stained in pink, while dead pollen cells are shown as empty and shrivellled shells. Scale bar=50 μm. Middle panels: examination of fresh pollen outer structure through scanning electron microscopy (SEM) analysis under low vacuum conditions. Affected mutants lines produced shrivelled and collapsed pollen grains. Scale bar=20 μm. Lower panels: transmission electron microscopy (TEM) of pollen sections revealed that the collapsed or shrivelled pollen grains are devoid of cytoplasm and organelles. Scale bar=10 μm.

Although no *ScFRK1* mRNA signal could be detected in mature pollen, a strong signal was observed in cross-sections of flower buds in the anthers (Fig. 2C, ix). The ~2mm flower buds corresponded to anthers where the sporogenous cells are differentiating into PMCs. Pollen from the *Scfrk1*-S27 and *Scfrk1*-S1 lines was used to pollinate a fully compatible *S. chacoense* genotype. When pollen from the WT (S-alleles S_12_S_14_, also used as the host for plant transformation) was used to pollinate this fully compatible genotype (S-alleles S_11_S_13_), 100% of the pollinated flowers developed fruits (*n*=20). When pollen from the *Scfrk1*-S27 line was used, only one in 20 pollinations led to the production of a fruit, while the use of pollen from the *Scfrk1*-S1 line did not lead to fruit production (*n*=20). This suggested that pollen development was also affected in transgenic plants down-regulated in *ScFRK1* mRNA levels. Pollen observation in dehiscent anthers revealed that, in transgenic lines, pollen viability was severely affected, as estimated by acetocarmine staining of >1000 pollen grains scored per line (Fig. 5, upper panels). Compared with the WT, where >98% of the pollen grains are stainable, <20% of the *Scfrk1*-S27 transgenic pollen and <1% of the *Scfrk1*-S1 transgenic pollen appeared viable. When fresh pollen is observed by SEM under low vacuum conditions, affected lines produced shrivelled and collapsed pollen (Fig. 5, middle panels). TEM of pollen sections revealed that the collapsed or shrivelled pollen grains were devoid of a dense cytoplasm and of organelles, in sharp contrast to WT pollen (Fig. 5, lower panels).

### Cytological analysis of microsporogenesis

**Fig. 6.**
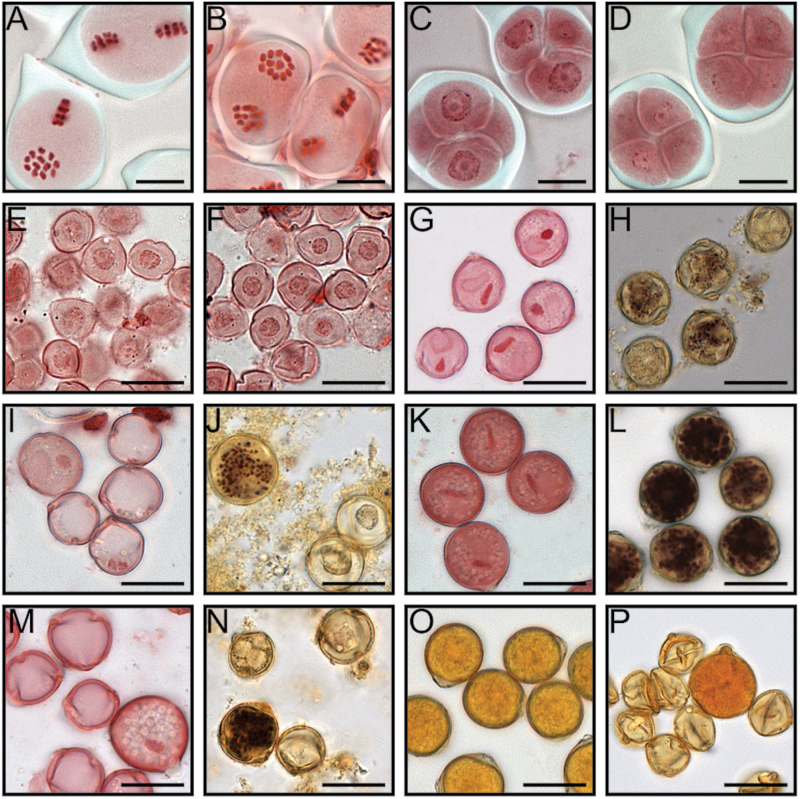
Comparative cytological analyses of WT and *Scfrk1*-S1 pollen. Late prophase II/metaphase II showing 12 chromosomes in WT (A) and in transgenics plants (B). Tetrads surrounded by callose from WT plants (C) and transgenics (D). Mononucleate microspores just released from the tetrads of WT plants (E) and transgenics (F). Young binucleate WT pollen stained with lacto-acetic orcein showing generative (dark) and vegetative (pale) nuclei (G), and initiation of starch accumulation, visualized with the iodine test (H). The same developmental stage as G and H in transgenics (I, J). WT pollen grains 3 d before anthesis (K, L). The same developmental stage as K and L in transgenics (M, N). Mature WT (O) and transgenic (P) pollen stained with iodine; by this time, starch hydrolysis has been completed. Note the collapsed pollen grains in the transgenic line surrounding one viable pollen grain (P). Scale bar=20 μm.

In order to determine precisely when the pollen started to collapse, a cytological analysis of developing pollen was conducted. Microscopic observations of PMCs both at late prophase II/metaphase II of meiosis (Fig. 6A, B) and at the tetrad stage (Fig. 6C, D) revealed no differences between the WT and the *Scfrk1*-S1 line. Similarly, the young mononucleate microspores from *Scfrk1*-S1 and the WT line appeared indistinguishable (Fig. 6E, F), suggesting that the defect occurred at later stages of development. This was indeed the case since at later stages of gametogenesis the two lines started to show substantial differences. Mitosis occurred normally in the microspores of the WT, and was followed by differentiation of the generative and vegetative nuclei (Fig. 6G). In contrast, in line *Scfrk1*-S1, <20% of the microspores underwent the first pollen mitosis (PMI), but <1% continued their development, leading to differentiation of the generative and vegetative nuclei (Fig. 6I). In *S. chacoense*, microsporogenesis proceeds similarly to the microsporogenesis reported in both tomato (de Nettancourt and Eriksson, 1968) and *S. verrucosum* (de Nettancourt and Dijstra, 1969). In these species, starch accumulation begins shortly after PMI, while starch hydrolysis begins 2 d before anthesis and is completed by the time the flower opens. In the present study, the iodine test was used to monitor starch accumulation and hydrolysis in developing pollen. The test revealed that almost all WT young pollen started to accumulate starch just after the pollen mitosis (Fig. 6H), and starch accumulation reached a maximum 3 d before anthesis. At this time, the pollen grains appeared almost black (Fig. 6L), and only the generative nucleus remained visible with the acetocarmine stain (Fig. 6K), the vegetative nucleus being completely hidden by the starch grains. In contrast, in *Scfrk1*-S1 pollen, starch started to accumulate only in a limited number (<1%) of the pollen grains (Fig. 6J), most probably in those where mitosis had been completed and differentiation of the generative and vegetative nuclei had occurred. Most probably these pollen grains continued their development in a similar way to the WT (Fig 6M, N). At anthesis, almost all WT pollen appeared viable, having completed starch hydrolysis (Fig. 6O). In line *Scfrk1*-S1, however, >99% of the pollen appeared shrunken, with only very few grains showing a normal appearance (Fig. 6P).

### Pollen tube guidance is severely affected in the *ScFRK1* transgenic plants

**Fig. 7.**
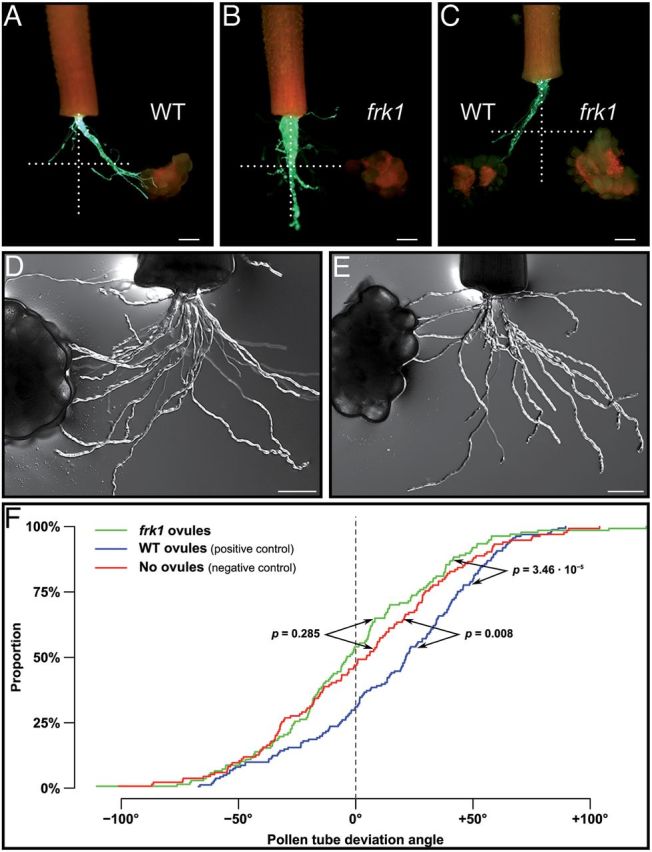
Pollen tube guidance is affected in *ScFRK1* transgenic plants. (A) Single-choice pollen tube guidance assay with WT *S. chacoense* ovules. Pollen tubes express the GFP marker under the control of the tomato Lat52 promoter (A–C). (B) Single-choice pollen tube guidance assay with *Scfrk1*-S1 ovules. (C) Two-choice pollen tube guidance assay with WT and *Scfrk1*-S1 ovules. (D, E) Single-choice pollen tube guidance assay with limited pollen load in order to measure pollen tube angles from WT (D) and *Scfrk1*-S1 ovules (E). (F) Kolmogorov–Smirnov statistical analysis from the angle distribution in D and E. Scale bar=200 μm.

The integrity of the embryo sac, the female gametophyte, has been shown to be a prerequisite for the ability of the ovule to attract pollen tubes. Mutants lacking a mature female gametophyte or affected in the development of its cells are defective in pollen tube guidance (reviewed in Marton and Dresselhaus, 2010; Chevalier *et al.*, 2011; Takeuchi and Higashiyama, 2011). To determine if the *ScFRK1* transgenic lines are also affected in pollen tube guidance, a semi *in vivo* pollen tube guidance system was used. The *Scfrk1*-S1 line was selected for this analysis as it showed the lowest percentage of functional embryo sacs. WT flowers were hand pollinated with fully compatible pollen and styles were collected 24h later. The detached styles are then laid on a microscopic slide covered with pollen tube growth medium with ovules placed at ~650 μm from the cut style end, a distance corresponding to the radius of the ovary. Pollen tubes start to emerge ~30 hours after pollination (HAP). Figure 7 shows the result of two different assay systems. First, a single-choice assay was used with ovules from either WT or *Scfrk1*-S1 plants as shown in Fig. 7A, B, respectively. Attraction was determined from the bulk pattern obtained, with each pollinated style being counted as one assay. An attraction phenotype was scored when there was a clear trend and the majority of the pollen tubes grew toward the ovules as observed in Fig. 7A with WT ovules, while typical absence of attraction can be observed in Fig. 7B with *Scfrk1*-S1 ovules. When WT ovules were used, 59% of the assays showed pollen tubes growing toward WT ovules (*n*=80), while only 10% of the pollen tubes grew toward the *Scfrk1*-S1 ovules (*n*=60), giving a highly significant *P*<0.0001 value in a two-sample binomial test. Furthermore, although 10% of the assays with the *Scfrk1*-S1 ovules showed pollen tubes growing toward them, pollen tubes never reached these ovules, in contrast to what is observed with WT ovules in Fig. 7A. Next, a two-choice assay system was used, with equidistant ovules from WT and *Scfrk1*-S1 plants (Fig. 7C). In this case, no difference in attraction between WT and *Scfrk1*-S1 ovules (null hypothesis) would lead to a 50:50 distribution. Out of 65 assays, 83% (54) showed clear attraction to WT ovules while 17% (11) grew toward *Scfrk1*-S1 ovules, which is highly significant in a one-sample binomial test (*P*<0.0001). As in the single-choice system, when pollen tubes grew toward *Scfrk*-S1 ovules, none reached the ovules. It is interesting to note that similar results were obtained in *Torenia fournieri*, where the investigators used a microfluidistic device to channel the pollen tubes toward a targeted ovary or a control (Horade *et al.*, 2013). To confirm the results of this quick assay system, the two-sample Kolmogorov–Smirnov test, a non-parametric test comparing empirical distribution functions in two samples, widely used in axon guidance studies, was also used. In this case, growth angles for all distinguishable pollen tubes were calculated from their exit to their end point on a total of ~150 pollen tubes from five semi *in vivo* single-choice assays (Fig. 7D–F). A mean angle of 2.5 ° was obtained for the negative control (without ovules), – 0.8 ° for assays with *Scfrk1*-S1 ovules, and 16.3 ° for assays with WT ovules. Attraction was thus observed with WT ovules (*P*=0.008) but not with *Scfrk1*-S1 ovules (*P*=0.285).

### Embryo sac-dependent gene expression and gametophytic to sporophytic communication

In order to isolate embryo sac-expressed genes and sporophytic genes that would depend on embryo sac gene expression, the *Scfrk1*-S1 ovule transcriptome was compared with the one from WT ovules using a 7.7K DNA microarray made from ovule-derived ESTs (Tebbji *et al.*, 2010). Analysis of variance (ANOVA) testing, along with a Benjamini and Hochberg multiple testing correction algorithm, was used to select ESTs that showed a statistically significant difference in transcript abundance between the WT and the *Scfrk1*-S1 ovules. A Welch’s *t*-test (*P*<0.05) was initially used to compare the profiles from the *Scfrk1*-S1 versus control and the control versus control comparisons. They were then further restricted with a ≥1.5 fold variation (1.5 cut-off up or down). Seventy-nine ESTs corresponding to 69 unigenes showed statistically lower transcript abundance between the WT and the *Scfrk1*-S1 ovules (Supplementary Table S1 at *JXB* online). These genes, identified as down-regulated in *Scfrk1*-S1 ovules, are thus most probably embryo sac-expressed genes. On the other hand, 118 ESTs (98 unigenes) were transcribed significantly more in *Scfrk1*-S1 ovules, and may thus be associated with sporophytic adaptation to the absence of female gametophyte (Supplementary Table S2).

Blast2GO was used to analyse functional category enrichment between up- and down-regulated genes, and a Fisher’s exact test was performed to determine which categories were significantly regulated (Supplementary Table S3 at *JXB* online). Among them, gene ontology (GO) terms related to chromatin remodelling (e.g. DNA packaging, DNA conformation change, histone exchange, nucleosome organization, and chromatin assembly and disassembly), cell cycle control (e.g. cell cycle, interphase), intracellular trafficking, as well as development of reproductive tissues characterized down-regulated genes. On the other hand, up-regulated genes are mostly associated with response to stress (e.g. defence response, systemic acquired resistance, response to cold and to radiation), senescence (ageing, organ senescence), and amino acid metabolism (e.g. serine family amino acid metabolic process).

SignalP 4.1, SecretomeP 1.0, and NetNGlyc 1.0 were also used to predict secretion and glycosylation of proteins encoded by regulated ESTs (Supplementary Fig. S3, Supplementary Table S4 at *JXB* online). A characteristic of embryo sac genes is their enrichment for small and secreted proteins (Jones-Rhoades *et al.*, 2007). Interestingly, proteins harbouring a signal peptide are significantly more frequent in down- and up-regulated genes than in unregulated genes (*P*=5.6e^–12^ and *P*=3.8e^–6^, respectively). In contrast, the proportion of unconventionally secreted proteins decreases significantly in both data sets (*P*=0.05 and *P*=0.02, respectively). Furthermore, 30% of secreted peptides potentially encoded in down and up ESTs correspond to small, cysteine-rich proteins (CRPs) such as defensins, lipid transfer proteins, rapid alkalinization factor (RALF) peptides, *Papaver* S-like proteins, and γ-thionins. Conventionally, up-regulated secreted proteins are significantly less glycosylated (*P*=0.03), which holds true for up-regulated CRPs (*P*=4.6e^–4^).

## Discussion

Among the protein kinases known to affect key aspects of plant reproductive development only a few have been characterized from the MAPK superfamily. These include the *Arabidopsis* YODA (MAPKKK4), involved in embryo and stomata development (Bergmann *et al.*, 2004; Lukowitz *et al.*, 2004); the *S. chacoense* FRK2 MAPKKK, involved in ovule (Gray-Mitsumune *et al.*, 2006) and pollen development (O’Brien *et al.*, 2007); the redundant *Arabidopsis* MAP3Kε1 (MAPKKK7) and MAP3Kε2 (MAPKKK6), involved in pollen viability (Chaiwongsar *et al.*, 2006); the *Arabidopsis* MPK6, involved in anther, inflorescence as well as in embryo development (Bush and Krysan, 2007); and *Arabidopsis* MPK3 and MPK6, also involved in defence responses and in ovule development (Wang *et al.*, 2008). Recently, *mpk3*/*mpk6* double mutant pollen tubes were also shown to be defective specifically in the funicular guidance phase (Guan *et al.*, 2014).

In this study, the characterization of ScFRK1, a novel MAPKKK from *S. chacoense* that affects both male and female gametophyte development, is reported. Although the three *FRK* genes, *ScFRK1*, *2*, and *3*, are expressed in reproductive tissues, they are not genetically redundant, since down-regulation of *ScFRK1* is not complemented by the presence of the others. They also share limited amino acid sequence identity, <45% (Supplementary Fig. S1 at *JXB* online), and are not all predicted to be in the same subcellular compartment (only ScFRK1 and 2 are predicted to be localized in the nucleus). Furthermore, an RNA interference effect on *ScFRK2* and *3* is unlikely considering the low nucleotide sequence identity between *ScFRK1* and the two other kinases (<38%) and the fact that no stretches of >15 and 12 identical nucleotides are found with *ScFRK2* and *ScFRK3*, respectively. Thus, as expected, *ScFRK2* and *3* expression levels were not significantly down-regulated in the *Scfrk1*-S1 line (Supplementary Fig. S2), confirming that the phenotype observed is due to the down-regulation of the *ScFRK1* gene. Furthermore, down-regulation of *ScFRK2* showed no observable phenotype, nor any reproductive defects, while overexpression lines led to the conversion of ovules into carpelloid structures (Gray-Mitsumune *et al.*, 2006).

As for *ScFRK2*, the other member of the family previously examined, *ScFRK1* had a complex and peculiar expression pattern. While *ScFRK2* expression is weak at anthesis and is fertilization induced, *ScFRK1* is strongly expressed at anthesis and is pollination repressed. Since *ScFRK1* is expressed early on during pollen and ovule development, with phenotypes observed in down-regulated lines affecting the gametophytes, high expression in the ovary at anthesis and the post-pollination steady-state *ScFRK1* mRNA decrease are puzzling and remain to be examined.

In *Arabidopsis*, the putative orthologues of the *Solanum* FRK family members are MAPKKK19, 20, and 21. Interestingly, MAPKKK20 was among the genes regulated by the male germline-specific DUO1 MYB transcription factor (Borg *et al.*, 2011). In contrast to *ScFRK1* transgenic lines, *DUO1* mutants only affect the male gametophyte. *DUO1* mutants progress normally through PMI but fail to complete the generative cell cycle (Durbarry *et al.*, 2005; Rotman *et al.*, 2005). In contrast, in line *Scfrk1*-S1, <20% of the microspores underwent PMI, and <1% continued their development, leading to differentiation of the generative and vegetative nuclei (Fig. 6I). Consistent with this, starch accumulation that normally begins shortly after PMI in solanaceous species (Fig. 6J) was only detected in <1% of the *Scfrk1*-S1 pollen grains (Fig. 6J), and those were presumably the ones that had progressed through PMI and completed the differentiation of the generative and vegetative nuclei. Thus, in pollen, *ScFRK1* most probably does not act in the same pathway or would act upstream of genes such as *DUO1*.

As *ScFRK1* is expressed in both sporophytic and gametophytic tissues, it is puzzling that only the gametophyte is affected in down-regulated transgenic lines. Considering that the ovule sporophytic tissue in *ScFRK1* transgenic lines does not show any evidence of developmental defects at maturity, and that in described gametophytic mutants sporophytic tissue develops normally (Bencivenga *et al.*, 2011), this suggests that down-regulation of *ScFRK1* mostly affects gametophyte development. Since very weak expression can still be detected in *ScFRK1* down-regulated lines, a different threshold effect between the expression observed in the integument and the young ovule at the MMC stage (see Fig. 2, vii for WT expression) could also explain why the sporophyte is not affected. Interestingly, *ScFRK1* expression is not equally distributed in the integument, with higher levels at the tip of the integument in young ovules at the MMC stage (Fig. 2, vii) and, in mature ovules, in the cell layers immediately surrounding the embryo sac, the inner epidermis, also called the ‘integumentary tapetum’ in unitegmic families such ase *Solanaceae* (Fig. 2iii, v). The inner epidermis has been endowed with numerous features. During the initial stages of gametophyte development, the ultrastructure of the inner epidermis cells has been described as akin to that of meristematic cells. By dividing profusely, its cells were considered to provide the necessary conditions to co-ordinate the intensive growth of the embryo sac. Once fully differentiated, the inner epidermis provides nutrition to the embryo sac (Kapil and Tiwari, 1978).

As expected from *Arabidopsis* mutants that lack a functional embryo sac (reviewed in Chevalier *et al.*, 2011; Takeuchi and Higashiyama, 2011; Dresselhaus and Franklin-Tong, 2013), or for plants where the synergid cells had been physically ablated (Higashiyama *et al.*, 2001), pollen tube guidance was severely compromised in the *Scfrk1*-S1 transgenic line (Fig. 7). Absence of the embryo sac led to the isolation of embryo sac-dependent genes (down-regulated genes; Supplementary Table S1 at *JXB* online) that could be directly involved in pollen tube guidance or other cell–cell interaction functions. Among these, two RALFs, ScRALF4 and 5, were isolated. These are closely related to *Arabidopsis* RALF27 and 32 and are quite ubiquitously expressed in *S. chacoense* (Germain *et al.*, 2005). No functions have yet been ascribed to these RALFs. However, involvement of RALF peptides in plant reproduction has been recently highlighted with the characterization of ScRALF3, involved in sporophyte to gametophyte communication. Although expressed in the sporophytic tissue of the ovule, down-regulation of *ScRALF3* expression by RNA interference led to improper embryo sac development through loss of embryo sac nuclei polarization and an increase in asynchronous divisions (Chevalier *et al.*, 2013). Absence of the embryo sac also had an impact on the surrounding sporophytic tissue with the isolation of several up-regulated genes in the ovule, suggesting interactions between the female gametophyte and the maternal sporophyte, as observed previously in *Arabidopsis* (Johnston *et al.*, 2007). Furthermore, the majority of proteins encoded in up and down ESTs correspond to secreted peptides, 30% of which are small CRPs. These proportions are in line with recent studies in *Arabidopsis* (Supplementary Figs S4 and S5).

Interestingly, the GO terms associated with down-regulated genes in *Scfrk1*-S1 (chromatin remodelling, cell cycle control, intracellular trafficking, and development of reproductive tissues) are consistent with the dynamic nature of the development of the embryo sac, with its rounds of mitosis, cellular polarization, and positioning, as well as with the expression of elevated amount of secreted proteins, such as from the synergid filiform apparatus. Similarly, GO terms associated with up-regulated genes are predominantly linked to stress responses, as if absence of the female gametophyte would be sensed as a scar and elicit a wound or defence response. The characterization of the other members of this MAPK cascade, such as MAPKK and MAPK, as well as downstream nuclear or cellular targets should reveal essential steps in male and female gametophytes development.

## Supplementary data

Supplementary data are available at *JXB* online.

Figure S1. (A) Section of a pMEKK phylogenetic tree showing the most closely related orthologues of ScFRK1–3 in *A. thaliana*. (B) Percentage sequence identity and similarity between *S. chacoense* FRK1, 2, and 3 and *A. thaliana* MAPKKK19, 20, and 21, based on a ClustalW multiple protein sequence alignment.

Figure S2. Specific down-regulation of *ScFRK1* in transgenic plants.

Figure S3. Secretion and glycosylation predictions for proteins up-, down- and not regulated in the *Scfrk1* mutant.

Figure S4. Conventional and unconvential secretion predictions for proteins regulated in *Scfrk1* and other ovule mutants.

Figure S5. CRP content in proteins regulated in *Scfrk1* and other ovule mutants.

Table S1. Information about *frk1* down-regulated ESTs.

Table S2. Information about *frk1* up-regulated ESTs.

Table S3. GO term enrichment in *frk1* up- and down-regulated ESTs compared with unregulated ESTs.

Table S4. Summary of secretion and glycosylation predictions on *Scfrk1* up-, down-, and unregulated ESTs.

Supplementary Data
