# Supplementary material for: The FRK1 mitogen-activated protein kinase kinase kinase (MAPKKK) from Solanum chacoense is involved in embryo sac and pollen development
Source: J Exp Bot. 2015 Jan 8;66(7):1833–43. doi: 10.1093/jxb/eru524 (PMC4378624; doi:10.1093/jxb/eru524)
Supplement: Supplementary Data [file supp_66_7_1833__index.html]

The FRK1 mitogen-activated protein kinase kinase kinase (MAPKKK) from Solanum chacoense is involved in embryo sac and pollen development — The FRK1 mitogen-activated protein kinase kinase kinase (MAPKKK) from Solanum chacoense is involved in embryo sac and pollen development — Supplementary Data 

# The FRK1 mitogen-activated protein kinase kinase kinase (MAPKKK) from *Solanum chacoense* is involved in embryo sac and pollen development

## Supplementary Data

Data files

**Files in this Data Supplement:**

- Supplementary Data - Supplementary Data
- Supplementary Data - Supplementary Data
