# Supplementary material for: The FRK1 mitogen-activated protein kinase kinase kinase (MAPKKK) from Solanum chacoense is involved in embryo sac and pollen development
Source: J Exp Bot. 2015 Jan 8;66(7):1833–43. doi: 10.1093/jxb/eru524 (PMC4378624; doi:10.1093/jxb/eru524)
Supplement: Supplementary Data [file supp_eru524_jexbot136390_file002.pdf]

The ScFRK1 MAPK kinase kinase (MAPKKK) from *Solanum chacoense* is involved in embryo sac and pollen development.

Edith Lafleur, Christelle Kapfer, Valentin Joly, Yang Liu, Faiza Tebbji, Caroline Daigle, Madoka Gray-Mitsumune, Mario Cappadocia, André Nantel and Daniel P. Matton

Supplementary material (Figures S1 to S5)

A

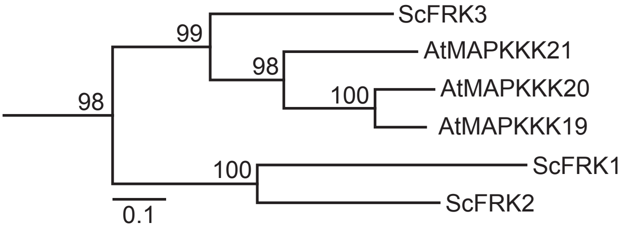

B

	FRK1	FRK2	FRK3	MAPKKK19	MAPKKK20	MAPKKK21
FRK1	100	44,0	30,9	33,4	34,4	33,8
FRK2	60,6	100	34,0	35,3	36,5	35,8
FRK3	45,6	47,0	100	45,7	46,0	48,7
MAPKKK19	45,6	47,9	59,8	100	74,9	58,5
MAPKKK20	46,4	49,1	57,2	85,3	100	55,8
MAPKKK21	45,6	49,1	63,2	70,1	68,4	100

% sequence identity

% sequence similarity

Figure S1. A. Section of a pMEKK phylogenetic tree showing the most closely related orthologs of ScFRK1 in *A. thaliana*. The phylogenic tree was inferred using a neighbor-joining algorithm on full-length protein sequences. Bootstrap support values from 1000 replicates are indicated beside the branches. The scale bar represents the expected number of substitution per site. B. Percentage of sequence identity and similarity between the *S. chacoense* FRK1, 2 and 3 and the *A. thaliana* MAPKKK19, 20 and 21, based on a ClustalW multiple protein sequence alignment.

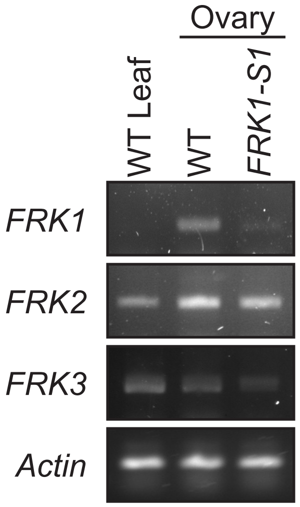

Figure S2: Specific down-regulation of *ScFRK1* in transgenic plants. RT-PCR analyses were conducted on WT leaf RNA, WT ovary RNA at anthesis and on *ScFRK1*-S1 mutant ovary RNA at anthesis for the three MAPKKK *ScFRK1*, *ScFRK2* and *ScFRK3* genes previously isolated (Germain et al., 2005; O'Brien et al., 2007; Tebbji et al., 2010). The actin gene was used as control. Twenty-five cycles were used for all samples.

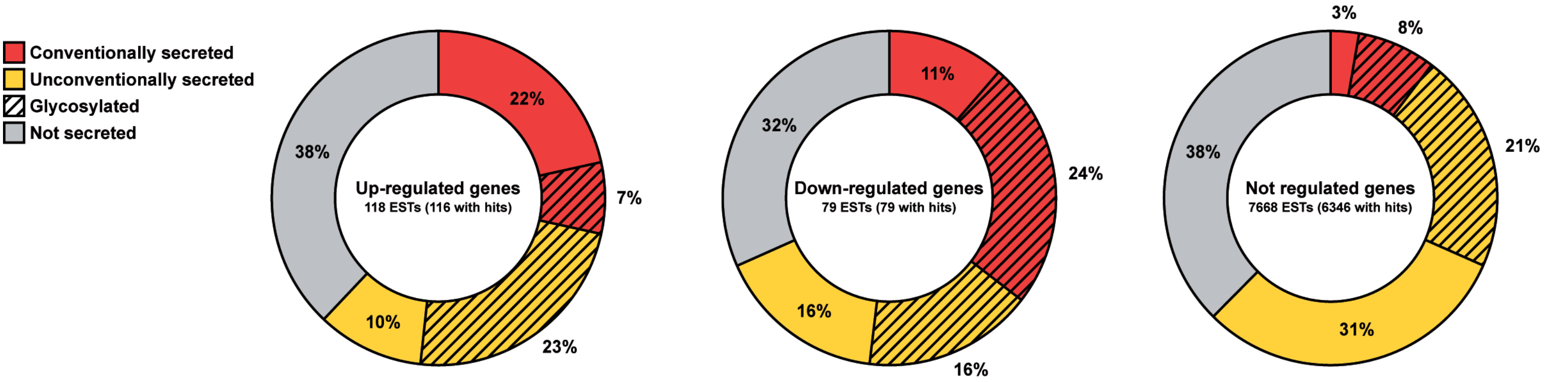

Figure S3. Secretion and glycosylation predictions for proteins up-, down- and not regulated in the *Scfrk1-S1* transgenic line. Conventional and unconventional secretion was predicted with the SignalP and SecretomeP programs, respectively. Proteins were considered unconventionally secreted when their SecretomeP score was above 0,5. Glycosylation was predicted with the NetNGlyc 1.0 program. Proteins were considered glycosylated if at least one asparagine was predicted to be glycosylated with a score greater than or equal to "+".

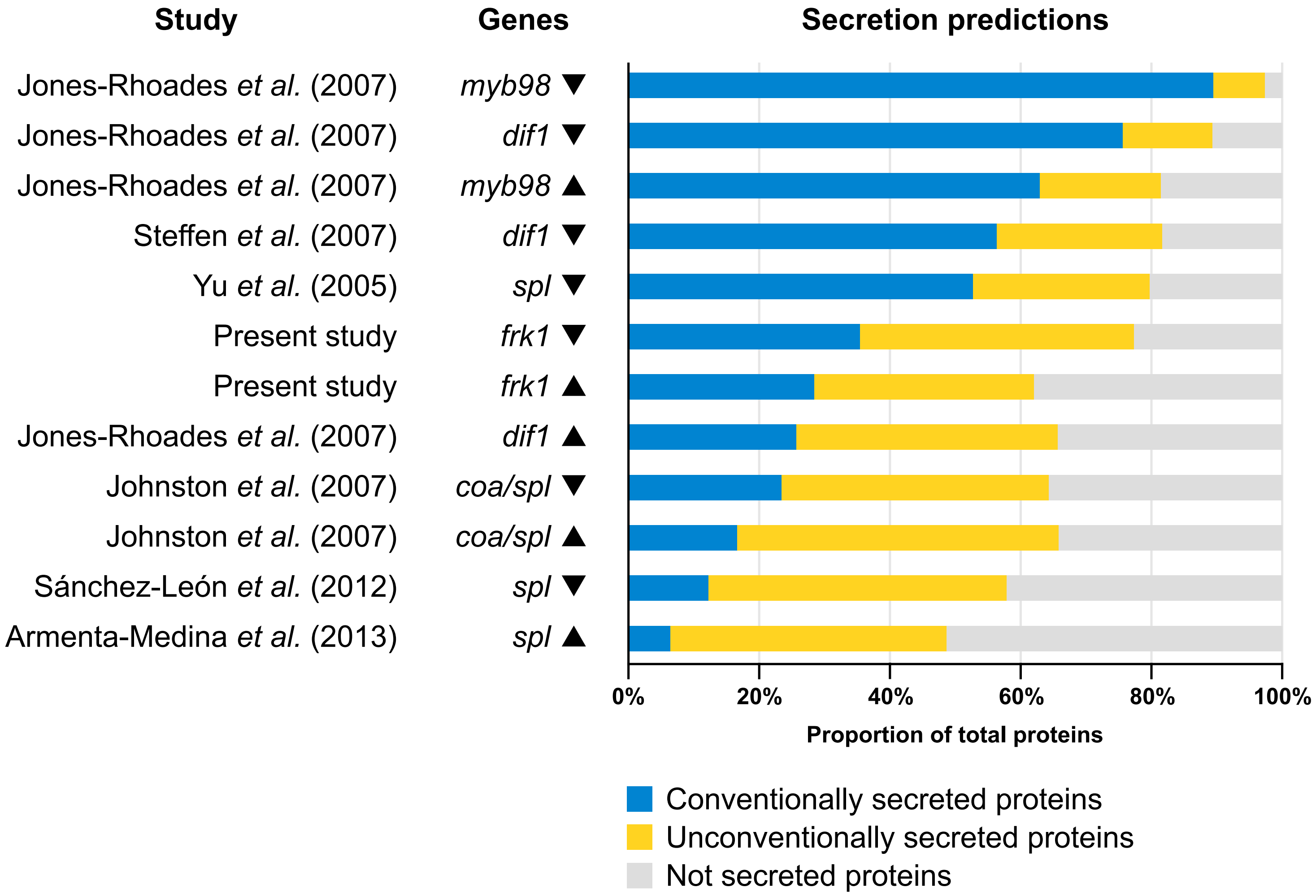

Figure S4. Conventional and unconvential secretion predictions for proteins regulated in *Sc-frk1-S1* line and other ovule mutants. Conventional and unconventional secretion were predicted with the SignalP and SecretomeP programs, respectively. Proteins were considered unconventionally secreted when their SecretomeP score was above 0,5.

References:

Armenta-Medina A, Huanca-Mamani W, Sanchez-Leon N, Rodriguez-Arevalo I, Vielle-Calzada JP. 2013. Functional analysis of sporophytic transcripts repressed by the female gametophyte in the ovule of Arabidopsis thaliana. PLoS One 8, e76977.

Johnston AJ, Meier P, Gheyselinck J, Wuest SE, Federer M, Schlagenhauf E, Becker JD, Grossniklaus U. 2007. Genetic subtraction profiling identifies genes essential for Arabidopsis reproduction and reveals interaction between the female gametophyte and the maternal sporophyte. Genome Biol 8, R204.

Jones-Rhoades MW, Borevitz JO, Preuss D. 2007. Genome-wide expression profiling of the Arabidopsis female gametophyte identifies families of small, secreted proteins. PLoS Genet 3, 1848-1861.

Sanchez-Leon N, Arteaga-Vazquez M, Alvarez-Mejia C, Mendiola-Soto J, Duran-Figueroa N, Rodriguez-Leal D, Rodriguez-Arevalo I, Garcia-Campayo V, Garcia-Aguilar M, Olmedo-Monfil V, Arteaga-Sanchez M, de la Vega OM, Nobuta K, Vemaraju K, Meyers BC, Vielle-Calzada JP. 2012. Transcriptional analysis of the Arabidopsis ovule by massively parallel signature sequencing. J Exp Bot 63, 3829-3842.

Steffen JG, Kang IH, Macfarlane J, Drews GN. 2007. Identification of genes expressed in the Arabidopsis female gametophyte. Plant J 51, 281-292.

Yu HJ, Hogan P, Sundaresan V. 2005. Analysis of the female gametophyte transcriptome of Arabidopsis by comparative expression profiling. Plant Physiol 139, 1853-1869.

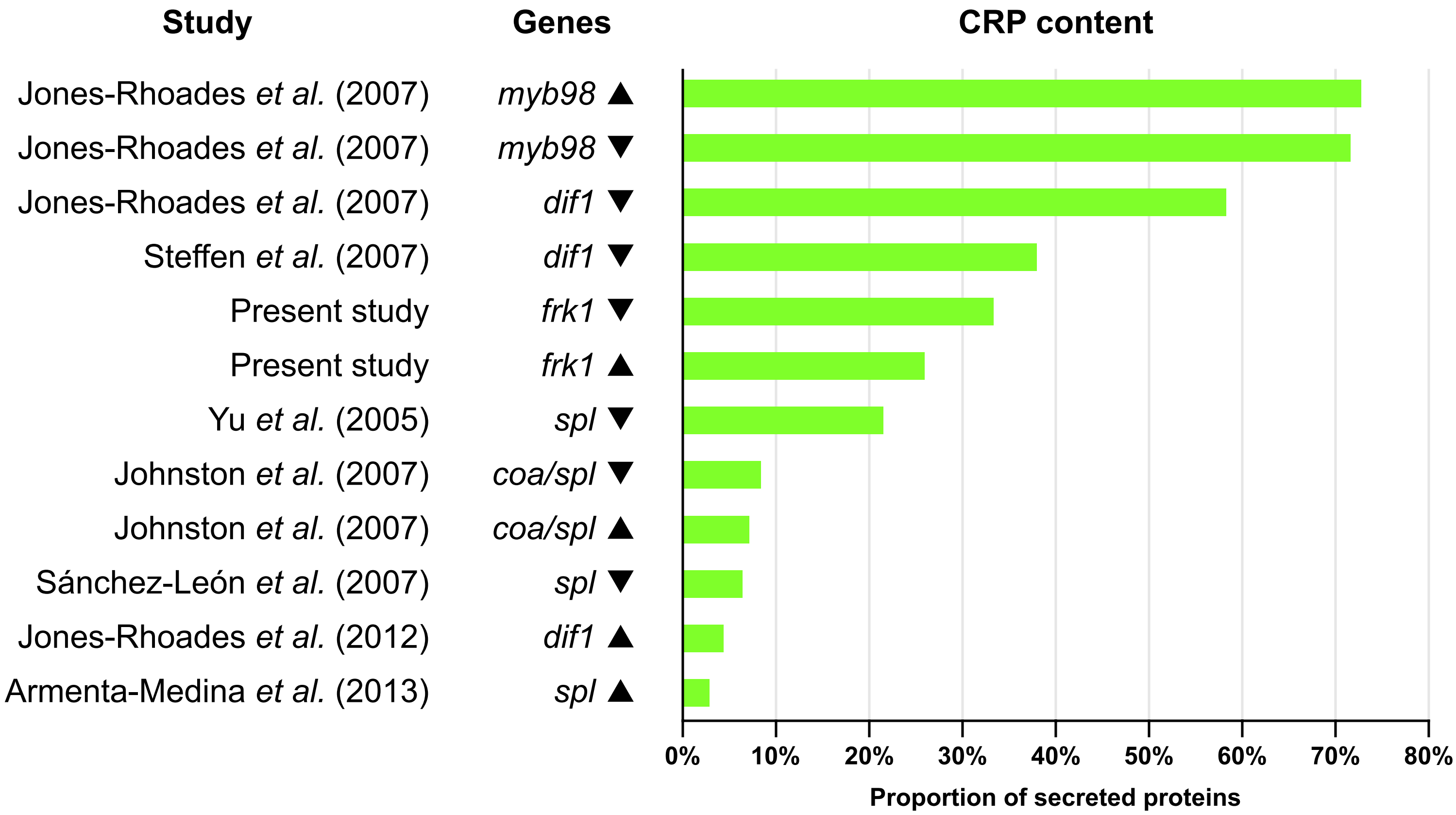

Figure S5. CRP content in proteins regulated in *Scfrk1-S1 line* and other ovule mutants. Proteins were considered as CRPs if they had 200 aminoacids or less, including 6 cysteines or more, and a conventional signal peptide or an unconventional secretion peptide prediction.
